# A case report of thrombolysis resistance: thrombus ultrastructure in an ischemic stroke patient

**DOI:** 10.1186/s12883-020-01706-3

**Published:** 2020-04-14

**Authors:** Ye Li, Heying Wang, Lili Zhao, Yating Jian, Meijuan Dang, Yu Jiang, Yiheng Zhang, Lei Zhang, Huqing Wang, Ru Zhang, Mingxia Chen, Guilian Zhang

**Affiliations:** 1 Department of Neurology|, the Second Affiliated Hospital of Xi’an Jiaotong University, No. 157 Xiwulu, Xi’an, 710004 Shaanxi China; 2 Electron Microscopy Center, Department of Medicine, Xi’an Jiaotong University, Xi’an, 710049 Shaanxi China

**Keywords:** Thrombus, Ultrastructure, Thrombolysis resistance, Mechanism

## Abstract

**Background:**

Following acute ischemic stroke (AIS), approximately half of patients do not achieve recanalization after intravenous administration of tissue plasminogen activator (rt-PA). Thrombolysis resistance is a possible reason for recanalization failure. Thrombolysis resistance is likely related to the ultrastructure and composition of the thrombus. However, there is a paucity of published information on the relationship between thrombus ultrastructure and thrombolysis resistance.

**Case presentation:**

Two patients who underwent mechanical thrombectomy were observed within 4.5 h after stroke onset. One patient failed to respond to rt-PA (defined as thrombolysis resistant), and the other patient did not receive rt-PA treatment (non-rtPA). In each patient, the occluded artery was the internal carotid artery or middle cerebral artery. According to the Trial of ORG 10172 in Acute Stroke Treatment classification, both patients had large atherosclerotic cerebral infarction. By scanning electron microscopy (SEM) and transmission electron microscopy (TEM), we found that the thrombus structure was significantly different between the two patients.

**Conclusion:**

Grid-like dense fibrin, compressed polyhedral erythrocytes, and large accumulation of neutrophils may be characteristics of thrombolysis resistant thrombi.

## Background

Stroke is the third cause of death worldwide and the main cause of chronic, severe adult disability [1]. Acute ischemic stroke (AIS) accounts for approximately 80% of stroke cases. Rapid recanalization is the basis of successful treatment. At present, thrombectomy is the recommended first-line treatment for large vessel occlusion, but intravenous tissue plasminogen activator (rt-PA) is still the preferred treatment for patients with an AIS event of less than 4.5 h prior. However, reperfusion is successful in less than 50% of patients who receive intravenous rt-PA. The reasons for reperfusion failure include (i) the characteristics of thrombolytic drugs, (ii) excessive thrombus overload or insufficient dose of thrombolysis drugs, (iii) the location of the thrombus, (iv) an unfavorable amount of time following onset, (v) non-fresh blood clots, and (vi) thrombolytic drug resistance, which may be one of the most important reasons for the failure of thrombolysis recanalization [2, 3]. Nonetheless, there is no clear mechanistic explanation for thrombolysis resistance. We hypothesized that analysis of the structure and character of resistant thrombi would illuminate the basis for thrombolysis resistance. Thus, we compared the ultrastructure of a thrombus that failed to yield to rt-PA with a thrombus from a patient who did not receive rt-PA therapy.

## Case presentation

A 65-year-old male patient (weight 56 kg) presented to our department with left limb weakness, slurred speech, and deviated mouth that began 4 h prior. The patient had a history of smoking, diabetes, hyperlipidemia, and coronary atherosclerotic heart disease. Thrombolysis was initiated with 50 mg rt-PA 1.5 h after onset when hemorrhage was not found by head computer tomography (CT). After rt-PA, the patient had intermittent unconsciousness, slurred speech, right gaze, left facial paralysis, and left limb paralysis. The power in his left limbs was grade 0. The National Institutes of Health Stroke Scale score was 19. Brain digital subtraction angiography (DSA) showed immediately: (i) ophthalmic artery segment of right internal carotid artery (RICA) completely occluded and grade II collateral circulation was established; (ii) about 60% extracranial stenosis of the RICA; (iii) the left internal carotid artery was stenosed about 30% at the ophthalmic artery segment. After communicating with patient family, the patient underwent thrombectomy in RICA. Five clusters of 2 × 4 mm dark red thrombi were retrieved. These thrombi were stored immediately in an electron microscope fixing solution at low temperature. After 30 min, the DSA showed that the blood vessels were not re-occluded, and the RICA system had a forward blood flow of level III.

A second patient, an 83-year-old female, arrived in our department with right limb weakness that started 4 h prior. The patient had a history of coronary atherosclerotic heart disease. She had unconsciousness, left gaze, right facial paralysis, right limb paralysis (level 2), and a positive Babinski sign. The National Institutes of Health Stroke Scale score was 16. CT of the head did not show bleeding, but the family refused thrombolysis. DSA showed the trunk of left middle cerebral artery (MCA) occluded. The patient underwent left MCA thrombectomy. Three clusters of 1 × 2 mm dark red thrombi were retrieved (hereafter referred to as the *non-rtPA thrombus*). DSA showed that the forward blood flow was grade III in the left MCA.

The patients had histories of coronary atherosclerotic heart disease for 11 and 7 years, respectively. They underwent coronary stenting prior to stroke, and accepted aspirin and atorvastatin calcium continuously. During hospitalization, they underwent 72 h cardiac rhythm detection and ultrasonic cardiography to exclude atrial fibrillation and mural thrombus. Low-density lipoprotein cholesterin was 4.2 mmol/L, and glycosylated hemoglobin was 6.8%. No other abnormalities were evident from laboratory blood tests that included blood count, renal and hepatic function tests, blood glucose, myocardial enzymes, coagulation, and troponin.

**Fig. 1:**
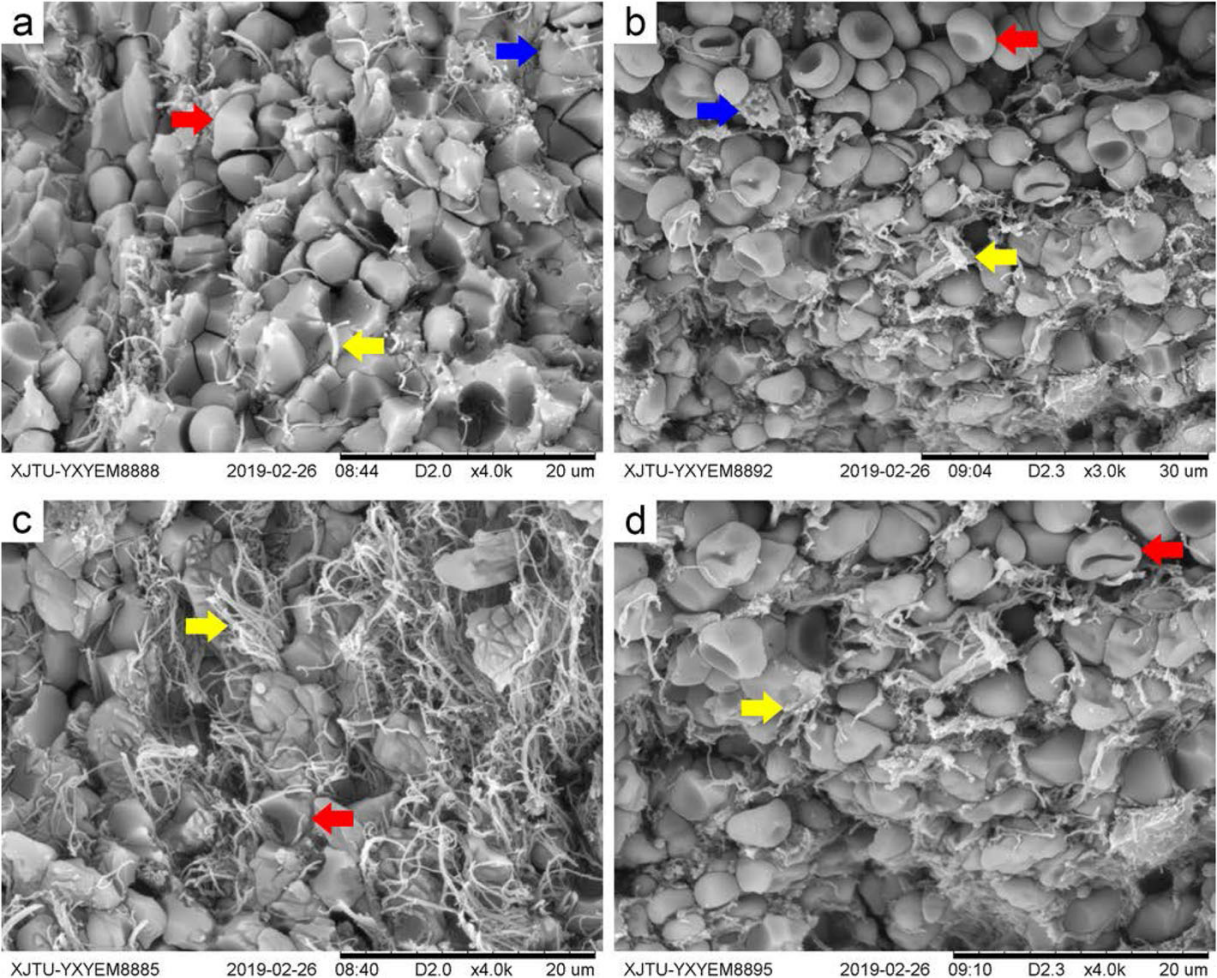
SEM of the rt-PA (**a**, **c**) and the non-rtPA thrombus (**b**, **d**). The red arrow indicates erythrocytes, blue is platelets, and yellow is fibrin

Scanning electron microscopy (SEM) showed the following thrombus features: (i) The fibrin arrangement was different in the two thrombi. In the rt-PA thrombus, fibrin had increased and ruptured, and it was arranged mainly in a grid pattern. In the non-rtPA thrombus, the fibrin was sparse, intact in structure, and irregular in arrangement. (ii) The erythrocyte and platelet morphologies were different in the two kinds of thrombi. The rt-PA thrombus lacked the erythrocyte double concave discs and platelet polyspinous processes, which were replaced by swollen, compressed polyhedral erythrocytes; platelets filled the fibrin grid. The non-rtPA thrombus exhibited normal erythrocyte and platelet morphologies, and cells were scattered in a disordered manner among the fibrin (Fig. 1).

**Fig. 2:**
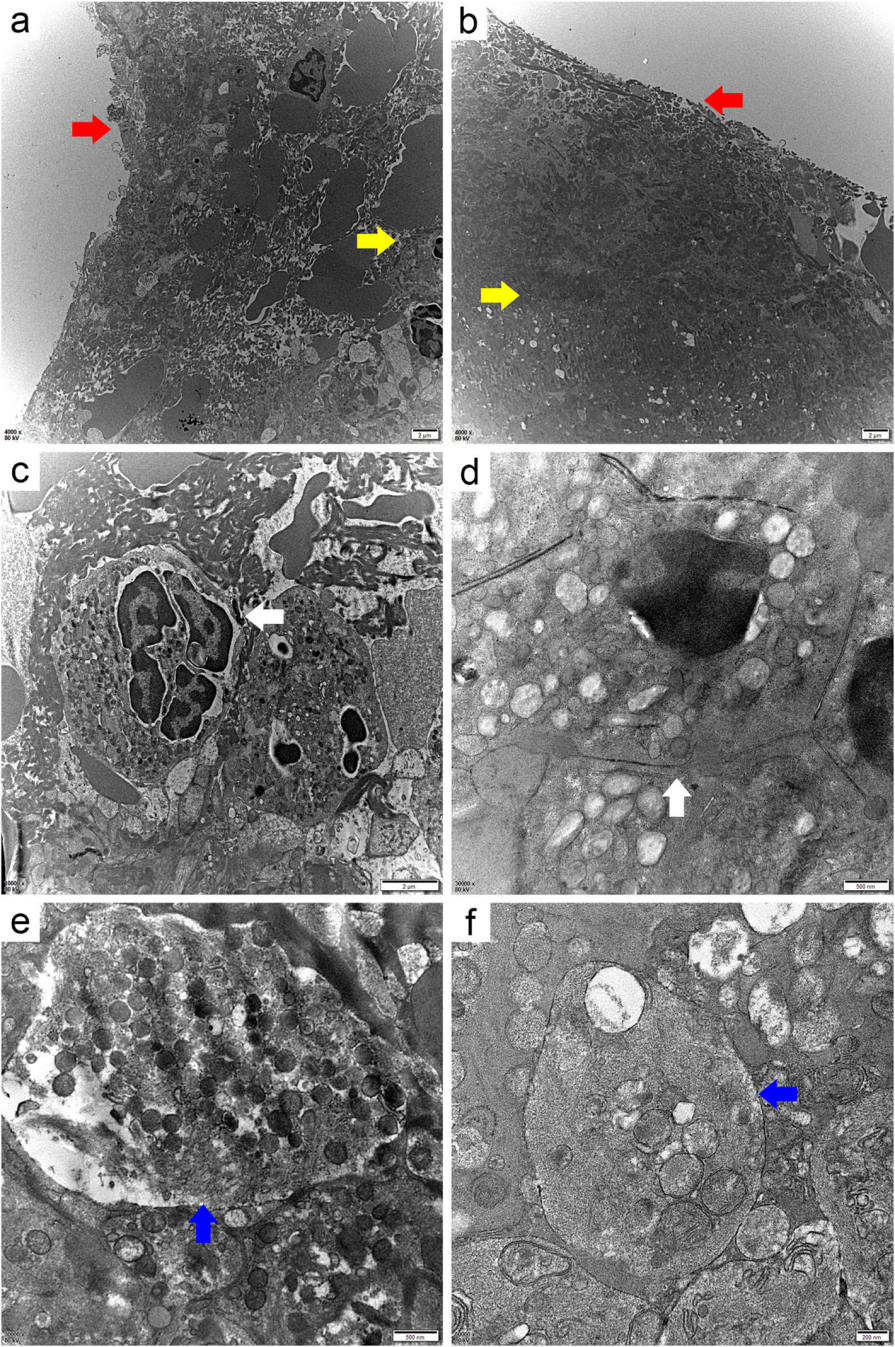
Comparisons of surface and internal structure between the rt-PA thrombus (**a**, **c**, **e**) and the non-rtPA thrombus (**b**, **d**, **f**) (TEM). The red arrow indicates the surface of the thrombus, the yellow arrow points to the inside of the thrombus, the blue points to platelets, and the white points to neutrophils. **a** shows that the thrombus surface is denser than the internal structure. **b** shows that the surface and internal structure of thrombus are similar in density. **c**, **d**, **e**, **f** show swollen platelets and mitochondria in both thrombi, which contained many vacuolar particles, spilled cellular contents, and indistinct structures

**Fig. 3:**
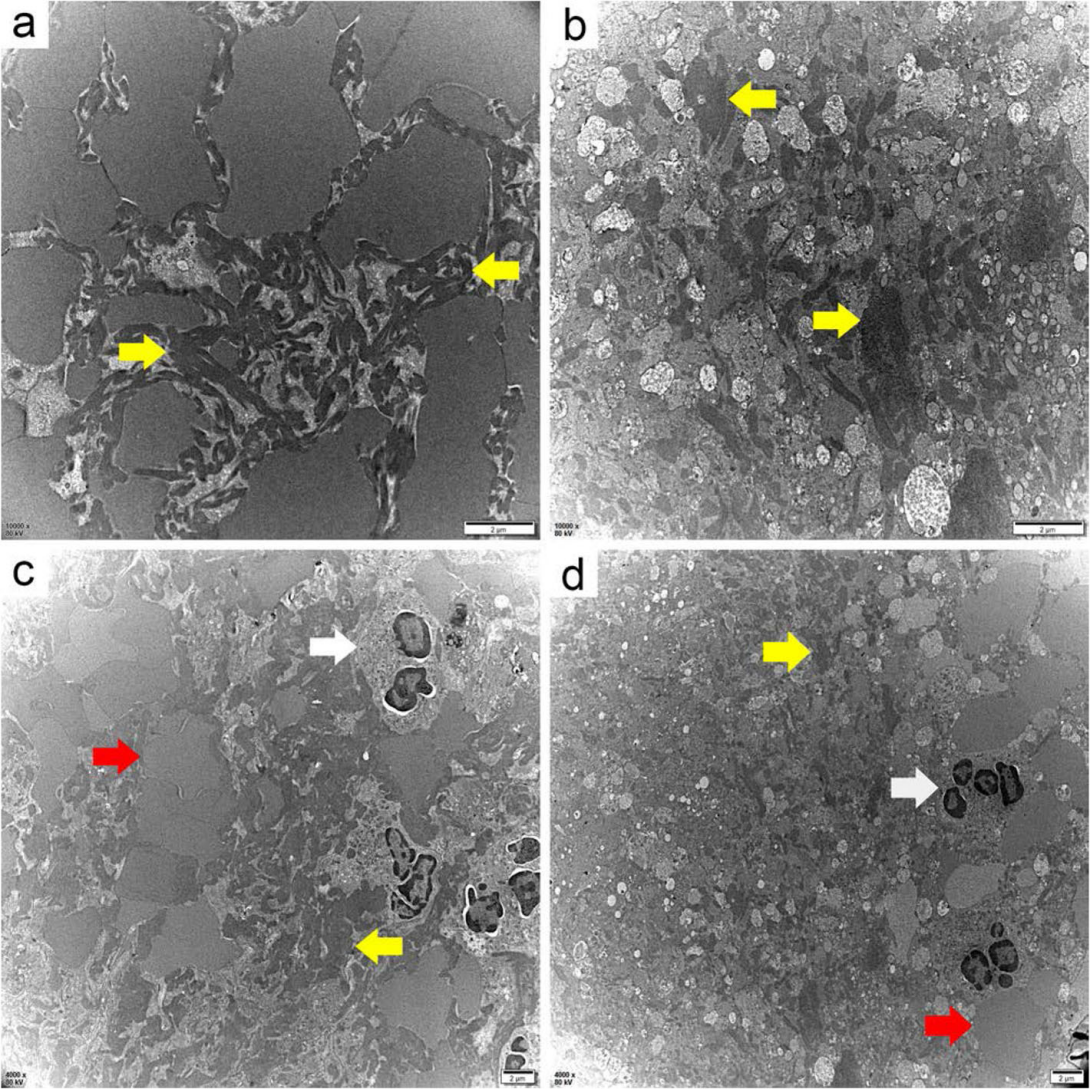
The internal structure of the rt-PA thrombus (**a**, **c**) and the non-rtPA thrombus (**b**, **d**) (TEM) **a**. **b** 10000 × 80 kV, **c**. **d** 4000 × 80 kV. The red arrow indicates erythrocytes, yellow points to fibrin, and white points to neutrophils. The fibrin morphology and arrangement, erythrocyte morphology, and granulocyte proportion were different for the two kinds of thrombi

Transmission electron microscopy (TEM) results likewise showed differences in thrombus structure: (i) The two thrombi had different fibrin morphologies and arrangements. The fibrin in the rt-PA thrombus was fine, dense, and bundled, whereas in the non-rtPA thrombus fibrin was coarse, loose, and irregular. (ii) The erythrocyte morphologies were different in the two kinds of thrombi. The erythrocyte disc had disappeared, and the cell volume became larger in the rt-PA thrombus compared with in non-rtPA thrombus. (iii) Swollen platelets and mitochondria occurred in both kinds of thrombi, which contained many vacuolar particles, spilled cellular contents, and indistinct structure. (iv) The proportion of lobulated nucleated cells was different between the two types of thrombi. In multiple random fields, there were clustered and more lobulated nucleated cells with only a few erythrocytes and fibers in the rt-PA thrombus compared with the non-rtPA thrombus. (v) The surface and internal structures of the two thrombi had different densities. The surface structure of the rt-PA thrombus was denser than the interior, whereas there was no significant difference between the internal and surface densities of the non-rtPA thrombus (Figs. 2 and 3).

## Discussion and conclusions

Development of thrombectomy has made possible in vitro study of human cerebral thrombi. Thus, we used SEM and TEM to assess the structure of thrombi from two AIS patients. One patient failed to respond to rt-PA, and the other patient did not receive rt-PA. According to the Trial of ORG 10172 in Acute Stroke Treatment classification, both patients had a large atherosclerotic cerebral infarction. We found that the structure of the thrombus differed significantly between the two patients. In the non-rtPA thrombus, erythrocyte morphology was normal; the tightness between internal structures and surface structures did not differ noticeably. In the rt-PA thrombus, erythrocytes presented as close-packed polyhedra, and the surface structures were tighter compared with the internal structures.

Staessens et al. [4] reported that, if the section was intact, each segment within a thrombus had the same elements, including fibrin, erythrocytes, platelets, and von Willebrand factor. This indicated that any segment of the thrombus could express the overall characteristics. In our study, we randomly selected multiple fields to observe the thrombus at the same time. Our results were consistent with the report by Staessens et al. Cines et al. [5] found that blood clots underwent continuous contraction over time in vitro. There was a tight fibrin network and aggregated platelets in the surface of the contracted clot, and compressed polyhedral erythrocytes were found within the clot. Di Meglio et al. [3] used SEM to analyze a thrombolysis resistant clot; they found a thick, compact outer shell surrounding a loose erythrocyte-rich core, a structure similar to the structure of the resistant thrombus in our study. These findings raise additional, yet unanswered questions. What is the dense outer structure? Is the tight external thrombus structure and loose inner structure related to thrombolysis resistance?

Other physical and chemical properties could affect thrombus lysis. Almekhlafi et al. [6] found that there was a fine powder-like distribution of calcium embedded in the thrombolysis resistant thrombi, which suggested calcific thrombi might render rt-PA less effective. The fiber diameter is another possible factor. The rt-PA binds more broadly and moves faster in thick fiber clots. The thicker the fiber, the more rapid is thrombolysis [7]. We found that the fibers in non-rtPA thrombus were thicker than the fibers in the rt-PA thrombus, which may explain the difference in dissolution outcome.

Hosokawa et al. [8] found that plasma plasminogen activator inhibitor-1 (PAI-1), soluble vascular adhesion molecule-1, and P-selectin were significantly elevated in AIS patients who had thrombolysis failure. These blood biomarkers were obviously related to the recanalization after thrombolysis. The following are possible mechanisms: (i) Direct inhibition of thrombolysis: PAI-1 can bind rapidly to the active site of plasminogen activator, form an inactive complex of intracellular metabolism, block plasminogen activation, and inhibit thrombolysis [9]. In addition, thrombin activated fibrinolysis inhibitor (TAFI) removes residues from fibrin undergoing degradation, which eliminates plasminogen binding sites and inhibits thrombolytic action [10]. (ii) Individual variation: Genetic polymorphisms of fibrinolysis inhibitors TAFI and PAI-1 may affect rtPA-induced revascularization in AIS patients [9]. (iii) Fibrin structural changes cause a denser thrombus: The free radical-modified fibrinogen and thrombin form a dense clot structure that is insoluble in plasmin, which prevents exposure of the plasmin cleavage site [11]. (iv) Changes in other thrombus components, e.g., rich platelets and especially neutrophil extracellular traps (NETs) composed of a special fibrous network derived from neutrophils [12]. Ducroux et al. [13] reported that NETs mainly surround the thrombus, forming a scaffold-like structure that may promote rt-PA resistance. We found a greater number of neutrophils in the rt-PA thrombus, which could have contributed to thrombolysis resistance, a speculation worthy of further research.

Our study had several limitations. First, we were unable to confirm whether the non-rtPA patient had thrombolysis resistance. Second, there was no strong evidence that the differences we observed were responsible for thrombolysis resistance.

In conclusion, the mechanism of thrombolysis resistance is unclear. The resistant thrombus had a grid-like dense fibrin arrangement, compressed polyhedral erythrocytes, and a large accumulation of neutrophils, but further research is needed to distinguish the exact mechanism of resistance.

## Data Availability

Data are contained within the manuscript.

## Abbreviations

AIS: acute ischemic stroke
rt-PA: recombinant tissue plasminogen activator
SEM: scanning electron microscopy
TEM: transmission electron microscopy
CT: computed tomography
DSA: digital subtraction angiography
RICA: right internal carotid artery
MCA: middle cerebral artery
PAI-1: plasma plasminogen activator inhibitor-1
TAFI: thrombin activated fibrinolysis inhibitor
NETs: neutrophil extracellular traps
